# The Manchester Charcot Ankle Neuroarthropathy (M-CAN) Classification: A Radiographic-Based System for Assessing Charcot Ankle Neuroarthropathy

**DOI:** 10.7759/cureus.83099

**Published:** 2025-04-27

**Authors:** Kularaj Subramaniam, Iulia Valeria Rusu, Anand Pillai, Frank L Bowling

**Affiliations:** 1 Department of Trauma and Orthopedics, Hospital Tuanku Ja'afar, Seremban, MYS; 2 Department of Podiatric Surgery, University of Manchester, Manchester, GBR; 3 Department of Trauma and Orthopedics, Wythenshawe Hospital, Manchester University NHS Foundation Trust, Manchester, GBR; 4 Developmental Biomedicine Research Group, Department of Podiatric Surgery, University of Manchester, Manchester, GBR

**Keywords:** ankle, bone loss, charcot neuroarthropathy, eichenholtz, foot perfusion, glycemic control, hba1c

## Abstract

Background

Charcot neuroarthropathy (CN) is a complication of long-standing, poorly controlled diabetes, associated with increased risks of amputation and mortality. Currently, there is no dedicated classification system specifically for ankle CN, nor a standardized description of the possible deformities involving the ankle in relation to CN. This study aims to develop and propose the Manchester Charcot Ankle Neuroarthropathy (M-CAN) classification - a descriptive, radiograph-based system designed to categorize ankle deformities and patterns of bone loss in patients with diabetic CN. The M-CAN classification may aid clinicians in early identification of deformities, facilitating more effective treatment planning and improved outcomes.

Methods

This retrospective review included 71 patients with diabetic ankle CN who were managed at the Manchester University NHS Foundation Trust over a 10-year period. Patterns of ankle deformity and bone loss were assessed using weight-bearing radiographs, and this information formed the basis for developing the classification system. The proposed classification is structured as follows: “A” refers to the alignment of the ankle joint - Type 1: varus ankle, Type 2: valgus ankle, Type 3: anterior angulation, Type 4: posterior angulation, Type 5: combined plane deformity, and Type N: neutral ankle. “B” denotes bone loss around the ankle joint - Subtype a: tibial bone loss, Subtype b: talar bone loss, Subtype c: calcaneal bone loss, and Subtype d: combined bone loss. “C” represents the cutaneous condition around the ankle, including ulceration and infection. “D” reflects diabetic control, measured by glycated hemoglobin levels. “E” captures the modified Eichenholtz stage of Charcot - Stages 0, 1, 2, and 3. “F” indicates foot perfusion status as assessed by Doppler studies. The classification primarily describes the deformity pattern “A”, bone loss “B”, and CN stage “E” using standard weight-bearing ankle radiographs, with optional supplementary information from the cutaneous condition “C”, glycemic control “D”, and pedal perfusion status “F”.

Results

A total of 75 ankle X-rays from CN patients were reviewed. The coronal and sagittal planes of deformity “A” were assessed, and the patterns of bone loss “B” were documented, along with the Eichenholtz stage “E”. Based on this A+B+E framework, the most common classification among patients was Type 1-d Stage 3, indicating a varus ankle with combined tibial, talar, and calcaneal bone loss in the consolidation stage of CN. Inter- and intra-observer agreement for components A and B showed near-perfect reliability, with a Kappa value of 0.94.

Conclusions

This new classification system for ankle CN facilitates the understanding of deformity patterns using plain radiographs and serves as a descriptive tool. It allows the condition to be categorized based on the specific deformities and associated osseous loss.

## Introduction

Charcot neuroarthropathy (CN), also known as osteo-neuroarthropathy, is a well-documented complication most commonly associated with poorly controlled diabetes. The incidence is estimated at three to eight cases per 1,000 individuals with diabetes per year [1]. Treatment options range from conservative management to surgical interventions and, in severe cases, amputation. Due to its complexity and high risk of infection and treatment failure, CN imposes a significant financial burden on healthcare systems worldwide.

The underlying pathology of CN is primarily explained by the “neuro-traumatic” and “neuro-inflammatory” theories [2]. Despite these established mechanisms, the true incidence and prevalence remain unclear. For example, based on data from the Danish National Patient Registry, the prevalence of CN among individuals with diabetes in Denmark is 0.56%, with an incidence of 7.4 per 10,000 [3]. Other regions report varying prevalence rates: a study from Southeast Ireland found a prevalence of 0.26%, while data from the East Midlands of England showed a lower rate of 0.04% [1]. These variations suggest that many cases remain undiagnosed or unreported.

Several staging and classification systems have been developed to characterize CN. Eichenholtz [4] introduced a temporal and radiographic-based classification, dividing the condition into distinct neuropathic stages. Stage 1, or the fragmentation phase, is characterized by joint disorganization, bony debris, fragmentation, and periarticular fractures, often leading to joint laxity or subluxation. Stage 2, the coalescence phase, involves resorption of bone fragments and new bone formation seen on radiographs. Stage 3, the reconstruction or resolution phase, is marked by stabilization of the bone structure, with or without residual deformity. In 1990, Shibata [5] added a prodromal stage (Stage 0), defined by clinical signs such as warmth, swelling, and redness, with minimal or no radiographic changes, detectable primarily via MRI.

Other classification systems have been proposed by Brodsky [6], Sanders and Frykberg [7], and Schon et al. [8], but these are broader and include both foot and ankle CN. Currently, there is no specific classification that details deformity patterns or bone loss exclusively related to ankle CN. This gap may be attributed to the condition’s relatively low incidence of three to eight per 1,000 diabetic individuals per year [9,10] and the general lack of early recognition or clinical experience with ankle-specific CN presentations.

We propose that a standardized classification framework is essential to improve communication among healthcare professionals, simplify clinical documentation, and support strategic treatment planning. The aim of the new classification - the Manchester Charcot Ankle Neuroarthropathy (M-CAN) system - is to enhance the categorization of ankle CN and contribute to a better overall understanding and management, whether surgical or conservative. Currently, there is no widely accepted system describing the deformities and bone loss in ankle CN using plain weight-bearing radiographs. The M-CAN classification addresses this gap, enabling clinicians to consistently define the type and severity of ankle deformity. This consistency is critical for effective multidisciplinary communication, planning appropriate treatment strategies, and facilitating future research in this complex condition.

## Materials and methods

A retrospective review was conducted on patients diagnosed with CN under the care of the Manchester University NHS Foundation Trust (MFT) over a 10-year period. Most of these patients presented with clinically apparent deformities that altered hindfoot alignment and led to gait abnormalities. Clinical findings from follow-ups, serial radiographs, and weight-bearing ankle X-rays of the affected limb were manually recorded and compiled using Microsoft Excel (Microsoft Corporation, Redmond, WA, USA). Data were sourced from the Hive electronic patient record system and our dedicated foot and ankle Charcot database.

Inclusion criteria comprised patients with a confirmed diagnosis of ankle CN, availability of complete and high-quality weight-bearing ankle X-rays, adequate clinical documentation, and CN-related deformities involving the ankle. There were no restrictions on the duration of follow-up. Exclusion criteria included unconfirmed CN diagnosis, incomplete or poor-quality radiographs, insufficient documentation, other coexisting ankle pathologies, or previous surgery on the affected ankle.

A total of 75 affected ankles were identified among 71 patients, with four individuals presenting with bilateral ankle deformities. The cohort consisted of 48 male and 23 female patients, all of whom had been managed by a senior foot and ankle surgeon at MFT. All clinical and radiographic assessments, including the formulation of the classification system, were initially carried out by a foot and ankle fellowship trainee, with final verification by a senior consultant foot and ankle surgeon.

Radiographic parameters were assessed using weight-bearing AP and lateral X-rays to evaluate coronal and sagittal plane deformities of the ankle. Bone loss involving the tibia, talus, and calcaneus, whether chronic or ongoing, was also assessed as part of the newly proposed classification system. Additionally, each case was staged according to the modified Eichenholtz system: Stage 1 (acute fragmentation or bony destruction), Stage 2 (bone resorption and/or new bone formation), and Stage 3 (remodeled bone with possible residual deformities). Staging was determined by a senior foot and ankle surgeon at our center.

We have named this new system the M-CAN classification. This radiograph-based framework enables the categorization of ankle CN without the need for advanced imaging techniques such as CT or MRI. The classification primarily relies on three key parameters - A, B, and E - to categorize all identified cases (Table 1). While the degree of bone loss could be further sub-classified, this was deemed unnecessary at the classification stage, as it would not contribute additional diagnostic value. However, such sub-classification may be useful in preoperative planning.

**Table 1:** M-CAN classification system, including the three main components, “A,” “B,” and “E,” which describe each of their variations ^*^ Leave unlabeled if no bone loss is present. ^#^ Bone loss can be further quantified based on a CT scan for preoperative planning by the treating surgeon. M-CAN: Manchester Charcot Ankle Neuroarthropathy

Component	Description	Type/subtype
“A” Alignment of the ankle	Varus	Type 1
	Valgus	Type 2
	Anterior	Type 3
	Posterior	Type 4
	Combined	Type 5
	Neutral	Type N
"B" Bone loss^*#^	Tibia	Subtype a
	Talus	Subtype b
	Calcaneum	Subtype c
	Combined	Subtype d
“E” Eichenholtz stage with Shibata modification	Stage 0 (Prodromal)	
	Stage 1 (Destruction)	
	Stage 2 (Coalescence)	
	Stage 3 (Consolidation)	

**Figure 1:**
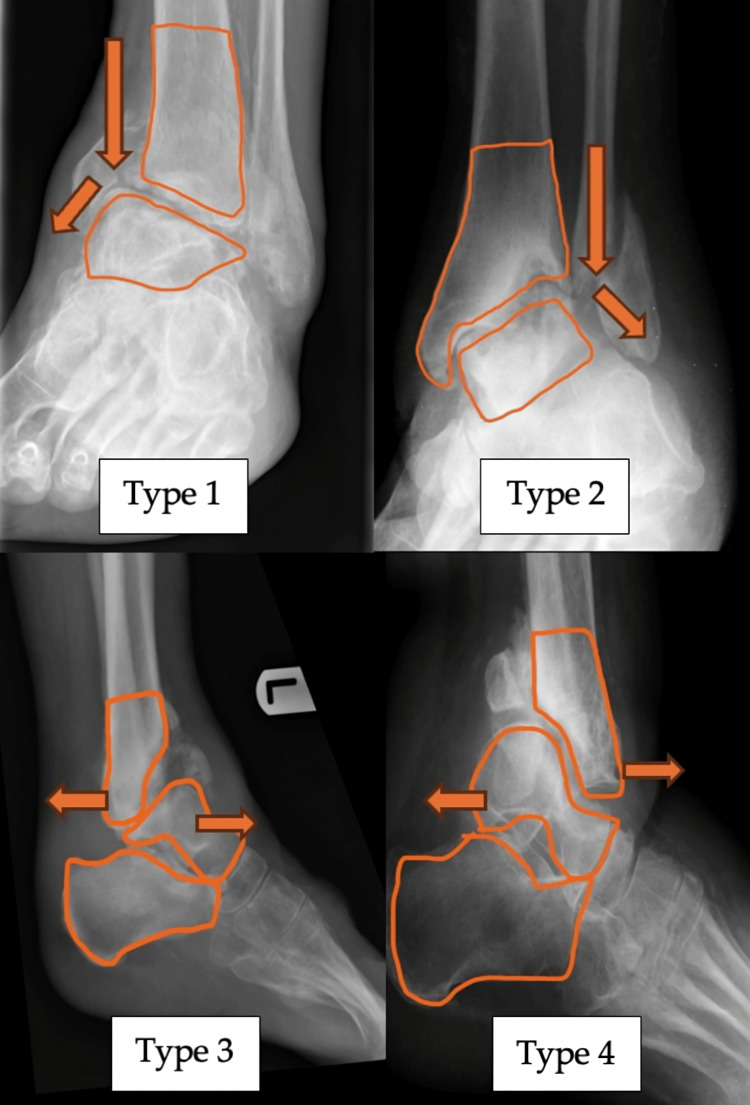
Radiograph with superimposed illustrations demonstrating four distinct alignment deformities associated with ankle CN Type 1: varus; Type 2: valgus; Type 3: anterior angulation; Type 4: posterior angulation of the ankle joint Arrows in the images indicate the direction of deforming forces, resulting in the respective deformities. CN: Charcot neuroarthropathy

In this system, “A” refers to the alignment of the ankle joint. As shown in Figure 1, the coronal plane deformities are categorized as Type 1 (varus) or Type 2 (valgus), while sagittal plane deformities are classified as Type 3 (anterior angulation) or Type 4 (posterior angulation). In cases with confirmed CN but no visible deformity, the alignment is designated as Type N (neutral). Multiplanar deformities, including subluxation or dislocation, are categorized as Type 5.

The coronal plane deformity was measured using the midline tibiotalar angle, the anatomical axis of the tibia, and the superior articular surface of the talus. Angles greater than 90 degrees were classified as valgus, while those less than 90 degrees were classified as varus [11]. For the sagittal plane, measurements were based on the lateral talar station, which defines the sagittal position of the talus relative to the anatomical tibial axis [12]. In a neutral position, the center of the talus lies within -0.81 mm to +3.15 mm of the tibial axis. All measurements were performed using Sectra UniView (Sectra AB, Linköping, Sweden), a universal medical image viewer available at the hospital.

**Figure 2:**
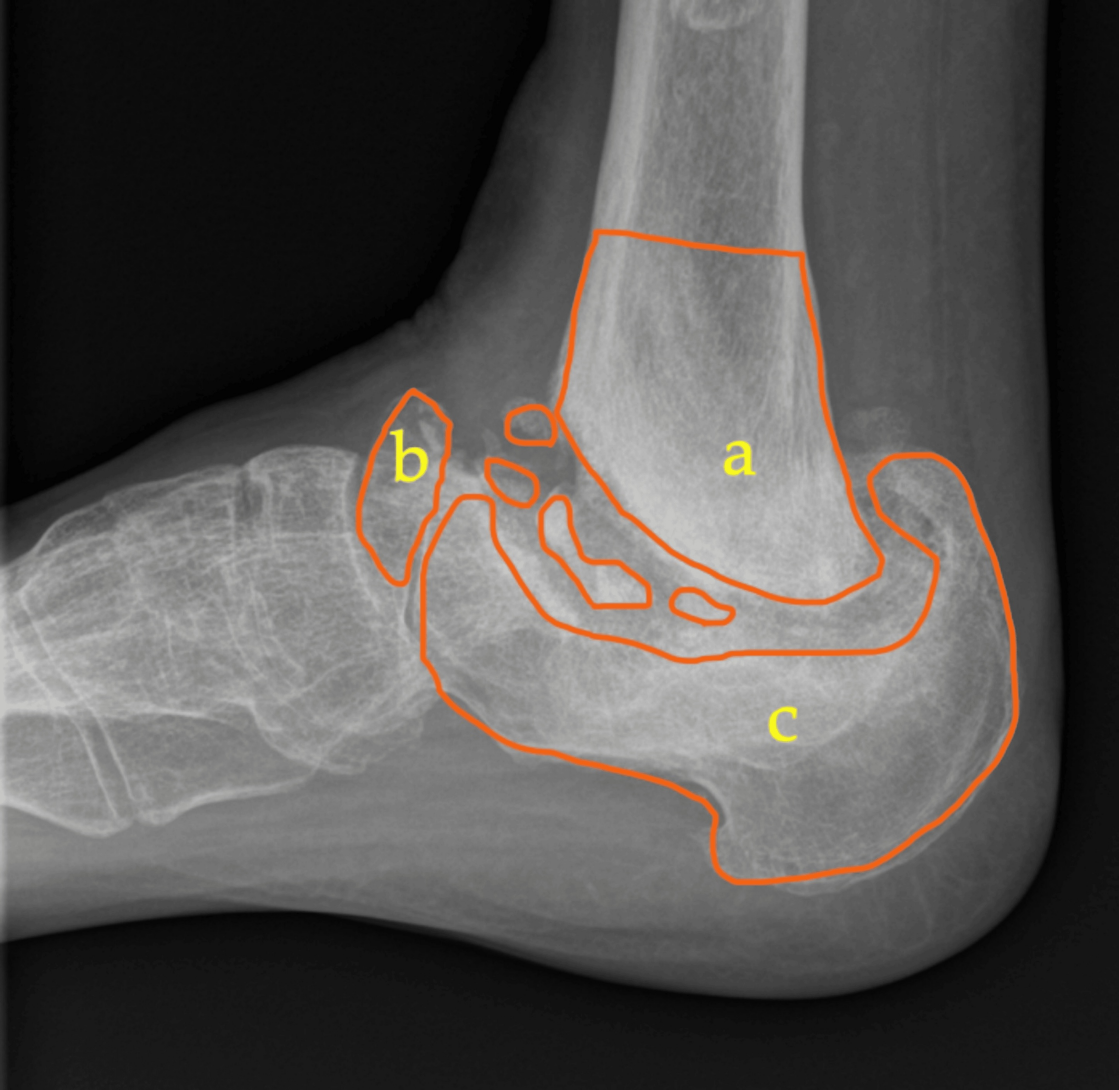
Radiograph depicting the anatomical sites of potential bone loss in ankle Charcot a: distal tibia; b: talus; c: calcaneum The extent of osseous loss may vary based on the chronicity of the condition.

Next, in the proposed classification, “B” represents bone stock, indicating the presence of bone loss, with subtypes a, b, c, and d. These subtypes correspond to bone loss in the tibia, talus, calcaneum, and a combined form, respectively. All patients in this review were classified based on plain weight-bearing ankle X-rays. Figure 2 illustrates the sagittal view of an ankle with bone loss involving the distal tibia and calcaneum, along with a severely fragmented talus.

We have also incorporated the modified Eichenholtz classification, denoted as “E,” into our system. The ankle Charcot can be classified from Stage 0 to Stage 3 at the time of examination. This staging serves as a guide for the timing and selection of treatment. Stages 1 to 3 can be assessed using plain X-rays, while Stage 0 may require additional MRI imaging if necessary.

The remaining components of the classification, “C,” “D,” and “F,” are included as supplementary elements to provide more comprehensive information (Table 2). These additional parameters evaluate the presence of an ulcer or infection, glycated hemoglobin (HbA1c) levels, and pedal perfusion status, all of which help determine the most appropriate treatment modality. Furthermore, they aid in predicting the outcomes of either conservative or operative interventions.

**Table 2:** M-CAN classification system, including the supplementary components “C,” “D,” and “F,” which enhance the comprehensiveness of the classification ^*^ Toe pressure can be used alternatively. HbA1c: glycated hemoglobin; M-CAN: Manchester Charcot Ankle Neuroarthropathy; PD: pulse diphasic; PM: pulse monophasic; PT: pulse triphasic

Component	Category	Subcategory
“C” Cutaneous condition	Ulcerated	Infected
		Non-infected
	Non-ulcerated	Infected
		Non-infected
“D” Diabetic control	Good control	HbA1c <53 mmol/L (<7.0%)
NICE Guidelines UK 2015	Poor control	HbA1c >53 mmol/L (>7.0%)
“F” Foot perfusion (pulse/Doppler)^*^	Triphasic PT	
	Diphasic PD	
	Monophasic PM	
	Pulseless	

The “C” component, which refers to the cutaneous condition, describes the skin condition of the involved ankle at the time of examination. The simplest classification is ulcerated versus non-ulcerated skin. Further details may include the presence or absence of infection, as determined through microbiological studies.

The next component, labelled as “D” for diabetic control, is crucial in the management of Charcot patients, especially those with diabetes. Often, the underlying disease control is not adequately addressed during the treatment of Charcot. However, the success of treatment is highly dependent on optimal disease control. The most straightforward monitoring tool for this is the measurement of HbA1c. Diabetic control is categorized based on whether the HbA1c level is below or above 53 mmol/L (7.0%). A patient with an HbA1c level within the accepted range is classified as “green,” while those exceeding this threshold are categorized as “red.” This parameter is critical, particularly if surgery is planned, as adequate diabetic control must be achieved. Multidisciplinary team involvement may be necessary to address and meet the acceptable HbA1c level.

The “F” component, for foot perfusion, refers to the assessment of foot perfusion at the time of examination. This can be evaluated in an outpatient setting using handheld Doppler scans. In the context of this classification system, PM refers to a monophasic pulse, PD to a diphasic pulse, and PT to a triphasic pulse. Both the anterior and posterior tibial arteries can be assessed, and the perfusion status should be recorded separately. If a pulse is absent, it should be documented as “pulseless.”

The proposed new classification system for ankle CN can be easily remembered using the simple mnemonic “ABCDEF.” The first three components, “A,” “B,” and “E,” are the key parameters to evaluate using plain X-rays. These parameters involve identifying ankle alignment deformities, associated bone loss, and the Charcot stage based on the modified Eichenholtz classification. The classification can be described as “A” + “B” + “E” = “ABE,” which encompasses the essential information derived from these components.

The supplementary components “C,” “D,” and “F” provide additional information that can guide treatment decisions and outcomes. By incorporating these additional elements, the classification can be further described as “ABE” + “C-D-F,” creating a more comprehensive assessment.

For example, a classification might be written as Type 1-d Stage 3, Ulcerated/Infected-Green-PM, which indicates a varus ankle with combined bone loss, the presence of an ulcer, a well-controlled HbA1c (below 53 mmol/L), and a monophasic pulse on Doppler studies.

We also conducted an intra-observer reliability study with five independent raters: three foot and ankle surgeons and two foot and ankle surgery fellows. This study spanned three months with X-ray reviews at two-week intervals, totaling six time points. Additionally, inter-observer reliability was evaluated among 20 independent raters, including three foot and ankle surgeons and 17 general orthopedic and trauma surgeons, in a single session during a monthly journal review meeting. The aim of these reliability assessments was to evaluate the raters’ ability to identify the plane of ankle deformity and associated bone loss based on plain weight-bearing X-rays. The raters were trained on the newly proposed classification system, with X-ray examples provided for each type of ankle alignment deformity (“A”) and each subtype of associated bone loss (“B”). The raters were then tasked with identifying the alignment deformities and associated bone loss, excluding the Eichenholtz stages of CN. Both intra- and inter-observer raters were blinded to each other’s findings.

## Results

Following a detailed review of the ankle X-rays in the AP, lateral, and oblique views, we classified a total of 71 patients, with 75 affected feet. Among these, 44 deformities were left-sided, and 31 were right-sided.

The mean age of the patients in this study was 60.5 ± 13.5 years. The population displayed a male predominance, with 48 males (67.6%) compared to 23 females (32.4%). Additionally, there was a higher prevalence of left-sided ankle CN (n = 44, 58.7%) compared to right-sided ankle CN (n = 31, 41.3%). Overall, the demographic profile indicates a middle-aged to elderly population, with a notable left-sided ankle CN and a male predominance. The demographic and clinical characteristics are summarized in Table 3.

**Table 3:** Demographic and clinical characteristics of the study population

Characteristic	Value
Age, years	
Mean ± SD	60.5 ± 13.5
Median	60
Range	28.0-91.0
Sex, n (%)	
Male	48 (67.6)
Female	23 (32.4)
Side, n (%)	
Left	44 (58.7)
Right	31 (41.3)

A total of 15 patients in the cohort were classified as Type 1-d, Stage 3, which included ankle varus deformity with a combined form of osteodestruction, involving either the distal tibia-talus or talus-calcaneum. Stage 3, as previously described, corresponds to the consolidation stage of Charcot, based on the modified Eichenholtz classification.

**Figure 3:**
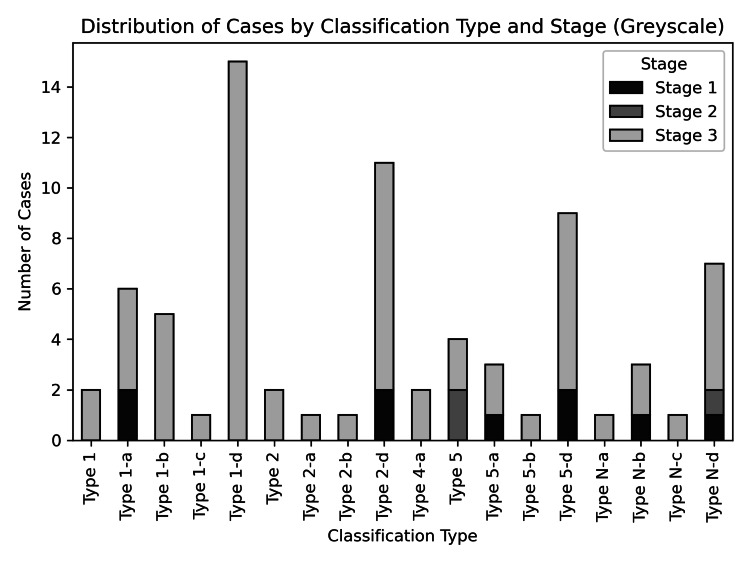
Case distribution according to types of deformity and stages of ankle CN CN: Charcot neuroarthropathy

Further analysis revealed that Type 2-d, Stage 3 represented the second largest group in the distribution, comprising nine ankles. This group included valgus ankle deformity with a combined form of osteodestruction in the consolidative phase of Charcot. The remaining patients were distributed across various other groups, as depicted in Table 4 and Figure 3.

**Table 4:** Total number of patients according to the types of deformity and stages of ankle CN CN: Charcot neuroarthropathy

Stage/type	Type 1	Type 1-a	Type 1-b	Type 1-c	Type 1-d	Type 2	Type 2-a	Type 2-b	Type 2-d	Type 4-a	Type 5	Type 5-a	Type 5-b	Type 5-d	Type N-a	Type N-b	Type N-c	Type N-d
Stage 1	0	2	0	0	0	0	0	0	2	0	0	1	0	2	0	1	0	1
Stage 2	0	0	0	0	0	0	0	0	0	0	2	0	0	0	0	0	0	1
Stage 3	2	4	5	1	15	2	1	1	9	2	2	2	1	7	1	2	1	5

In this review, we were able to identify the locations of bone loss involving the ankle and its adjacent joints. Both the distal tibia and talus exhibited nearly identical frequencies of bone loss. The bone loss may be isolated to either the distal tibia or talus, or it may involve the calcaneus. There are also combined forms of bone loss affecting the distal tibia-talus articulation, the talo-calcaneal articulation, and the entire hindfoot. These combined forms were the most prominent ones observed. Other forms of bone loss included the talo-navicular joint, anterior calcaneal process, posterior calcaneal tuberosity, and midfoot destruction.

In our cohort, all patients who presented to or were referred to our center were in Stages 1 to 3 of the modified Eichenholtz classification. The majority were in Stage 3, followed by Stage 1 and Stage 2. This classification allowed us to stratify and categorize them based on weight-bearing ankle X-rays, with no need for MRI, as it is primarily used to identify Stage 0 of Eichenholtz.

The primary defining parameters, “A” and “B,” were subsequently evaluated for inter-observer reliability by 20 independent raters: three foot and ankle surgeons and 17 general orthopedic and trauma surgeons. Intra-observer reliability was assessed among five independent raters: three foot and ankle surgeons and two fellow trainees in foot and ankle surgery. The Fleiss Kappa (κ) test was used to analyze reliability for both groups using IBM SPSS Statistics for Windows, Version 27 (Released 2020; IBM Corp., Armonk, NY, USA). The inter-observer reliability yielded a Kappa value of 0.948, indicating almost perfect agreement among observers. Similarly, the intra-observer Kappa value was 0.947. Agreement levels remained consistent across all evaluated X-rays, assessing ankle alignment deformity and associated bone loss. The modified Eichenholtz [13] staging was not tested for reliability, as its application by different observers can vary, leading to significant inconsistencies.

## Discussion

Chronic diabetic patients are particularly susceptible to developing CN in their fifth or sixth decade of life, with most having been diabetic for at least 10 years before the onset of CN [14]. Both type 1 diabetes mellitus and type 2 diabetes mellitus patients are at risk of fractures, as their bending resistance, trabecular stability, and stiffness are significantly reduced. Microarchitectural analysis shows increased cortical porosity, reduced cross-sectional bone area, and a low trabecular bone score [15]. The reduced bone density in diabetic patients with CN further increases their susceptibility to developing deformities, depending on the nature of the force or trauma affecting the involved ankle.

Several CN classifications have been proposed as fundamental guides for clinical assessment and the standardization of clinical research. Brodsky’s [6] classification system describes six anatomical sites, where the ankle is categorized as Type 3A. Sanders and Frykberg’s [7] system identifies five anatomical sites, with the ankle categorized as Type IV. Both systems emphasize that the anatomical sites of CN involvement determine the progression of the condition and its management, but neither delves into ankle CN in great detail. In contrast, the proposed M-CAN classification further elaborates on ankle CN by describing various forms of deformity and including the description of bone loss surrounding the ankle. This additional detail is crucial for understanding disease progression and potentially predicting outcomes.

The review of the “A” component, alignment, was performed to provide a better understanding of how and why the described deformities occur. The lateral ankle ligament complex is known to be the weakest link in terms of providing stability. In even the most trivial injuries, the anterior talofibular ligament fails, leading to ankle instability [16]. In the case of CN, repeated inflammation around the ankle compromises the integrity of the lateral ligament complex. Over time, this instability can lead to varus angulation and further osteodestruction. Our findings support this, as varus ankle angular deformity was the most commonly observed form in ankle CN. Understanding the plane of deformity is advantageous, as it aids in deciding the best surgical approach, particularly in the presence of a rigid deformity with contracted soft tissues.

Bone loss (“B”) or osteodestruction involving the Charcot ankle can be best explained through 3D CT bone morphometrical analysis, where the medial distal tibia is found to be more porous than the lateral distal tibia [17]. The reduced density in the medial distal tibia increases the likelihood of a break in the medial wall of the ankle fork, which can lead to the development of ankle varus deformity. This varus deformity may arise due to significant bone loss or resorption of the talus resulting from prolonged unprotected axial loading.

Physiological hindfoot alignment is typically characterized by a 0- to 5-degree valgus [18]. This is considered normal anatomy, where the ankle functions under axial loading. However, in the case of CN, this normal hindfoot valgus could be detrimental in the long term. With reduced bone mineral density in diabetic patients with CN, they are predisposed to fragility fractures [15,19]. Assuming the lateral ankle ligaments remain intact, the weakened bone over the medial ankle, coupled with the constant normal valgus loading of the hindfoot, would eventually lead to insufficiency or fragility fractures of the medial distal tibia or medial malleolus. Following these fractures and continued axial loading, the ankle would align with the anatomical hindfoot valgus, leading to valgus deformity of the joint. This could explain our observation of valgus ankle deformity as the second most common pattern in CN.

Various investigative methods have been described for diagnosing CN. Plain X-rays are sufficiently adequate for visualizing the plane of deformity and describing bone loss. Their widespread availability allows for the early identification of deformities and bone loss by general healthcare practitioners. MRI scans are crucial for differentiating acute CN from osteomyelitis, which may influence the decision for temporary or definitive stabilization [14,20]. CT scans, on the other hand, are useful in the later consolidated stages of the disease and can help quantify the extent of bone loss for preoperative planning and treatment staging [20]. We propose that the primary treating surgeon utilize CT or MRI as necessary, depending on the clinical situation.

The cutaneous condition (“C”) refers to the clinical appearance, which may present as an ulceration, either infected or non-infected. Understanding the cutaneous status is crucial, as it aids in determining the best treatment approach for a patient. If the ulcer is present but non-infected, treatment may involve an offloading cast to allow for healing, after which bony corrections can be offered. In cases of infected Charcot, treatment options may include intravenous antibiotics, serial debridement, staged reconstruction, or even amputation. Nilsen et al. reported an incidence of 64.9% of diabetic foot ulcerations in their study of Charcot patients, with 16.9% undergoing lower extremity amputations due to infected ulcers [21].

Incorporating “D” (diabetic control) through HbA1c levels in our classification serves as a reminder to assess the patient’s current diabetic control and reassess it after implementing appropriate measures. Achieving good glycemic control is crucial to preventing the progression of diabetic neuropathy and, consequently, CN. The NICE guideline recommends an HbA1c target of 48 mmol/mol (6.5%) [22]. Patients with poor control are at increased risk for ulcerations, with or without infection. Furthermore, poor glycemic control may impair or worsen surgical wound healing if surgical stabilization is necessary. A study on wound healing potential related to HbA1c control suggested that maintaining HbA1c levels between 7.0% and 8.0% promotes better wound healing and reduces mortality risks in diabetic foot ulcers [23].

In our review, we incorporated the modified Eichenholtz (“E”) stages to assist in decision-making regarding the most suitable treatment modality. Patients in Stage 0 of Eichenholtz typically exhibit no significant deformity at initial presentation. These patients benefit from total contact casting until remission is achieved. Stage 1 patients, however, experience a longer period of remission and are more likely to develop new deformities that may require surgery [24]. If diagnosis is delayed or if the condition progresses to later stages of Charcot, treatment options will differ and may become more costly and time-consuming.

The circulatory status of the foot, denoted as “F” (foot perfusion), is a crucial factor that should be evaluated. Müller et al. highlighted that peripheral arterial disease (PAD) is a significant contributor to delayed or impaired wound healing in foot and ankle surgery. Their study recommends a thorough preoperative evaluation of all patients undergoing foot and ankle surgery, including measuring the ankle-brachial pressure index [25]. Approximately 40% of patients with CN are likely to have PAD; however, the rate is significantly lower in patients with diabetic foot ulcers. The likelihood of ischemia development in CN patients is lower compared to those with diabetic foot problems or ulcers [26]. Nonetheless, it is important to assess the arterial status of these patients, as most severe deformities necessitate surgical stabilization. This allows for a better understanding of potential risks and complications related to wound healing and enables appropriate management strategies.

One of the key advantages of this classification system is that it enables the classification of ankle Charcot in various presenting forms using plain weight-bearing X-rays. This imaging modality is widely available and commonly used by healthcare practitioners, making it an ideal tool for diagnosing and monitoring Charcot cases. By facilitating early recognition of the condition, the system allows for timely referral to foot and ankle surgeons or trained personnel, ensuring that appropriate care is provided. Furthermore, this classification helps the receiving surgeon or specialist identify the pattern of deformity, providing insight into the mechanics of the presenting condition. Additional parameters, such as the cutaneous condition, HbA1c levels, and foot perfusion status, offer a comprehensive view that aids in selecting the appropriate treatment.

Recent studies indicate a growing lifetime prevalence of CN, with figures rising from 0.1% to 10% to an alarming 29-35% [27]. Misdiagnosis rates can reach as high as 79-95%, with treatment delays averaging around 29 weeks [28,29]. We hope that this classification system will help practitioners recognize patterns of deformity, prompting them to consider or diagnose ankle CN more effectively. This would enhance early detection and improve outcomes by preventing further progression of the deformity.

Limitations

We acknowledge some limitations in our review. We have not extensively discussed management strategies for each deformity type, and we intend to address this in future studies. Our classification primarily focuses on communicating the extent of the disease, assisting the treating surgeon in understanding the deformity pattern.

Another limitation is that our data were collected at a single institution, which may limit the generalizability of this classification system. To enhance the system’s applicability, future studies conducted across diverse healthcare settings and patient populations would be necessary to validate its broader use.

There is also the potential for observer bias, as the raters were briefed on the newly proposed classification. In real-world clinical settings, practitioners may not receive the same level of instruction, which could affect their ability to accurately apply the classification. However, given the lack of a previous classification system for ankle CN, it was essential to brief the raters on the new system to ensure they understood what to identify and document.

## Conclusions

The M-CAN classification is a reliable and descriptive system for characterizing ankle CN using plain radiographs. The development of this new classification supports clinical decision-making and enhances communication among healthcare providers. By understanding the patterns of deformity, clinicians can create more effective treatment plans, whether conservative or surgical. These patterns help identify the deforming forces that need to be addressed, potentially preventing further progression of the deformity and skin breakdown. Additionally, recognizing the osseous loss aids in planning the surgical approach and determining the type of fixation necessary to achieve a stable construct. A future prospective study on the M-CAN classification is warranted, with a focus on linking treatment strategies to each type of deformity and providing a prognostic guide for the management of ankle CN.
